# Copper-64 Chloride Exhibits Therapeutic Potential in Three-Dimensional Cellular Models of Prostate Cancer

**DOI:** 10.3389/fmolb.2020.609172

**Published:** 2020-12-01

**Authors:** Catarina I. G. Pinto, Sara Bucar, Vítor Alves, Alexandra Fonseca, Antero J. Abrunhosa, Cláudia L. da Silva, Joana F. Guerreiro, Filipa Mendes

**Affiliations:** ^1^Centro de Ciências e Tecnologias Nucleares, Instituto Superior Técnico, Universidade de Lisboa, Lisbon, Portugal; ^2^Departamento de Bioengenharia, iBB – Institute for Bioengineering and Biosciences, Instituto Superior Técnico, Universidade de Lisboa, Lisbon, Portugal; ^3^CIBIT/ICNAS Instituto de Ciências Nucleares Aplicadas à Saúde, Universidade de Coimbra, Coimbra, Portugal; ^4^Departamento de Engenharia e Ciências Nucleares, Instituto Superior Técnico, Universidade de Lisboa, Lisbon, Portugal

**Keywords:** copper-64 chloride, theranostics, prostate cancer, radiobiology, spheroids, cancer stem cells

## Abstract

Prostate cancer (PCa) is the second most common cancer type in men, and in advanced metastatic stages is considerable incurable. This justifies the need for efficient early diagnostic methods and novel therapies, particularly radiopharmaceuticals with the potential for simultaneous diagnosis and therapy (theranostics). We have previously demonstrated, using monolayer-cultured cells, that copper-64 chloride, a promising theranostic agent for PCa, has the potential to induce significant damage in cancer cells while having minimal side effects in healthy tissues. Here, we further explored this compound for its theranostic applications using more advanced PCa cellular models, specifically multicellular spheroids. Namely, we evaluated the cellular uptake of ^64^CuCl_2_ in three human PCa spheroids (derived from 22RV1, DU145, and LNCaP cells), and characterized the growth profile and viability of those spheroids as well as the clonogenic capacity of spheroid-derived cells after exposure to ^64^CuCl_2_. Furthermore, the populations of cancer stem cells (CSCs), known to be important for cancer resistance and recurrence, present in the spheroid models were also evaluated using two different markers (CD44 and CD117). ^64^CuCl_2_ was found to have significant detrimental effects in spheroids and spheroid-derived cells, being able to reduce their growth and impair the viability and reproductive ability of spheroids from both castration-resistant (22RV1 and DU145) and hormone-naïve PCa (LNCaP). Interestingly, resistance to ^64^CuCl_2_ treatment seemed to be related with the presence of a CSC population, since the most resistant spheroids, derived from the DU145 cell line, had the highest initial percentage of CSCs among the three cell lines under study. Altogether, these results clearly highlight the theranostic potential of ^64^CuCl_2_.

## Introduction

Prostate cancer (PCa)’s global incidence has been increasing over time, particularly in Asia, and Northern and Western Europe (Teoh et al., 2019), where it remains the second most common cancer type in men, posing a significant burden to healthcare systems (Bray et al., 2018; Teoh et al., 2019). Initial stages of PCa, when the tumor is organ-confined, are usually treatable; however, when PCa progresses to metastatic castration-resistant prostate cancer, the current treatments available, namely androgen deprivation therapy, become ineffective, leading to a much poorer prognosis (Hoang et al., 2017).

Copper is known to play important roles in cancer development and growth, having been shown to accumulate in several cancer types, including PCa (Gutfilen et al., 2018). This presents an opportunity that can be explored for cancer imaging and diagnosis through the use of medical radioisotopes of copper, of which ^64^Cu has been under the spotlight in recent years due to its many favorable characteristics (Boschi et al., 2018). Namely, it is suitable for both high resolution Positron Emission Tomography (PET) imaging, due to its positron (β^+^) emission, and for therapeutic purposes through the emission of β^–^ particles and Auger electrons (Boschi et al., 2018; Guerreiro et al., 2018). Clinical studies in humans have already been performed to investigate ^64^Cu biodistribution, dosimetry and lesion kinetics in patients with biochemical relapse of PCa, demonstrating ^64^CuCl_2_ effectiveness on the detection of local recurrence and bone and lymph nodes metastasis (Piccardo et al., 2018). This simplest form of ^64^Cu, which doesn’t require complexation with targeting ligands, is neither excreted via the urinary tract nor accumulated in the bladder, having a favorable biodistribution for detection of prostate tumors (Paparo et al., 2020). A kinetic analysis of PCa lesions showed that there is a rapid initial uptake of ^64^CuCl_2_, presenting a high tumor-to-background ratio after the first hour of administration, emphasizing its high diagnostic sensitivity in PET/CT (Righi et al., 2018). Recently, we have also showed for the first time that, in comparison to non-tumoral prostate cells, PCa cells exhibited higher ^64^Cu uptake after exposure to ^64^CuCl_2_. Furthermore, ^64^Cu ions were found to be able to reach the nucleus of tumor cells, inducing significant genotoxicity and cytotoxicity while causing less genetic damage in non-tumor cells. This could be explained by a deficient DNA-damage repair in PCa cells, which was in contrast with the non-tumor cells that were found to be able to efficiently repair the lesions induced by the radionuclide (Guerreiro et al., 2018).

Conventional *in vitro* cell culture systems fail to recapitulate some of the features of *in vivo* tumors, forcing cells to adapt to an unnatural growth conformation and leading them to lose some of the biological characteristics of the original tumor, such as differentiation, cell-to-cell interactions and extracellular matrix (ECM) contacts (Nath and Devi, 2016). Furthermore, drug evaluation assays performed in 2D culture systems may produce misleading results, due to differences in drug penetration and resistance mechanisms present in *in vivo* tumors, reinforcing the need to use more advanced culture systems prior to moving to *in vivo* animal testing (Kijanska et al., 2016; Ishiguro et al., 2017; Mittler et al., 2017). Multicellular tumor spheroids are one example of a 3D culture system where cells are driven to grow as spheres promoting cell-to-cell interactions and cell-to-ECM interactions (Zanoni et al., 2016), being able to re-establish the morphological, functional and mass-transport properties of *in vivo* tumors (Amaral et al., 2017), including the presence of cancer stem cells (CSCs) (Ishiguro et al., 2017). CSCs present in *in vivo* tumors, comprise a fraction of the cancer cell population that is able to generate the entire cancer structure due to their potential to self-renew and differentiate (Ishiguro et al., 2017), playing an important role in cancer relapse and metastasis formation due to their resistance to conventional chemotherapy and/or radiation therapy (Yu et al., 2012).

Studies using prostate spheroids and radionuclides have already been successfully performed, for example using radioimmunoconjugates (Ballangrud et al., 2001; Wang et al., 2006; Tesson et al., 2016), but none has yet tested the effects of ^64^CuCl_2_ in these advanced culture models. In this work, we aim to assess the effects of the exposure of human PCa multicellular tumor spheroids to ^64^CuCl_2_ in order to obtain new insights into some of the cellular consequences of exposure to this theranostic agent in advanced culture systems. For that, we have: (a) determined the presence of CSCs populations within the PCa spheroids; (b) evaluated the cellular uptake of ^64^CuCl_2_ in PCa spheroids obtained from 3 different cell lines; and (c) assessed the effects of exposure to this radionuclide on the PCa spheroids’ growth, viability, and clonogenic capacity.

## Materials and Methods

### Cell Lines and Media

Prostate cancer cell lines (22RV1, DU145, and LNCaP) were kindly provided by the Portuguese Institute of Oncology-Porto, Portugal. All cell lines were cultured in RPMI-1640 supplemented with 10% fetal bovine serum (FBS). Cell lines were grown at 37°C in a humidified atmosphere of 5% CO_2_ and tested for mycoplasma using the LookOut^®^ mycoplasma PCR Detection kit.

### Culture of Spheroids

Prior to spheroid formation, cells were cultured in monolayer on T25 or T75 culture flasks. When cells reached 80–90% confluence, cell suspensions with the desired cell concentration were prepared, and 200 μl of each cell suspension were seeded on each well of a Nunclon^TM^ Sphera^TM^ ultra-low attachment 96-well plate. The number of cells seeded per well was 2500, 3500, and 900 for 22RV1, DU145 and LNCaP, respectively, in order to obtain spheroids with a mean diameter in the range of 350–400 μm at day 3. The plate was then centrifuged at 405 *g* for 5 min and incubated at 37°C in a humidified atmosphere of 5% CO_2_.

### ^64^CuCl_2_ Solution Preparation

Copper-64 was produced in a medical cyclotron by irradiation of the ^64^Ni target as previously described (Alves et al., 2017) and supplied as a solution of ^64^CuCl_2_ in 0.1 M HCl. Prior to the biological studies, the pH of the solution was adjusted to ∼7 by adding appropriate volumes of 10 M NaOH, and 1 M phosphate buffer in order to avoid drastic changes in the culture medium pH upon addition of the ^64^CuCl_2_ solution.

### Flow Cytometry

Three-day old spheroids were pooled together, dissociated with Tryple, resuspended with PBS supplemented with 2% FBS (FACS buffer) and counted. The cell suspension was centrifuged at 1500 rpm for 5 min and resuspended with FACS buffer at a concentration of 1 × 10^6^ cells/ml. All subsequent steps were performed in the dark at room temperature. 1 × 10^5^ cells/tube were first incubated with Far Red LIVE/DEAD Fixable Dead Cell Stain Kit (Thermo Fisher Scientific) according to the manufacturer instructions to assess cell viability. After washing with 2 ml of FACS buffer, the cell pellet was resuspended with 100 μl of FACS buffer and surface stained with CD44 FITC (clone BJ18, Biolegend) or CD117 FITC (clone 1004D2, Biolegend) anti-human antibodies by incubation at room temperature for 15 min. After a washing step, cells were fixed with 100 μl of 2% formaldehyde at 4°C for 15 min, washed and resuspended with FACS buffer. Flow cytometry was performed on a FACSCalibur cytometer (BD Biosciences) and data was analyzed using FlowJo v10 software (FlowJo LLC).

### ^64^CuCl_2_ Cellular Uptake Assay

Three-day old PCa spheroids were used for the cellular uptake assay. A total of 100 μl of culture medium were removed from each well, and 100 μl of a solution of 0.9–1.9 MBq/ml of ^64^CuCl_2_ (in culture medium) were added to the spheroids, which were then incubated for 1, 2, and 3 h at 37°C. At each time point, for each cell line, 7 spheroids were pooled in a microtube, centrifuged at 1500 rpm for 3 min and the supernatant was removed. The cell pellet was washed with 500 μl of PBS, before cells were lysed in 500 μl of 1 M NaOH through incubation at 37°C for 10 min. The radioactivity associated with the cells was measured using a gamma counter (CRC^®^-55t, Capintec) and the uptake was calculated as the percentage of total activity normalized to the mean area of the spheroids of each cell line and to the maximum uptake measured at 1 h (in LNCaP spheroids). Three independent assays were performed for each cell line.

### Spheroids’ Growth, Circularity and Viability Determination

Spheroids’ growth was monitored every other day, starting at day 1, using a Primovert Inverted ZEISS Microscope (under 40× total magnification), with an integrated HDcam camera, connected to a computer with the ZEN 2 (blue edition) software. The spheroids’ area was determined using SpheroidSizer, a software available online (Chen et al., 2014). The circularity of the spheroids was determined according to (1).

(1) $$\text{Circularity}=\frac{4\,\pi \times \text{Area}}{{\text{Perimeter}}^{2}}$$

For the monitoring of spheroids exposed to ^64^CuCl_2_, 3-day old spheroids were exposed (or not, as a control) to 0, 0.6, 1.1, and 2.8 MBq of ^64^CuCl_2_ and grown for 8 additional days. After 4 days of exposure (7th day of spheroid growth), half of the medium was replaced by fresh medium, and at the 8th day of exposure (11th day of culture), the spheroids’ viability was measured using the acid phosphatase (APH) assay. Briefly, spheroids were washed with PBS through partial medium removal. After partial removal of the washing solution, 100 μl of APH buffer (0.1 M sodium acetate, 0.1% triton X-100 (vol/vol), and 2 mg/ml p-nitrophenylphosphate at pH 4.8) were added to the wells. After incubation at 37°C for 90 min, 10 μl of 1 M NaOH were added to each well, and the absorbance was measured at 405 nm using a microplate reader (Power Wave Xs, Bio-Tek). Empty wells were used as blank controls. Three independent experiments were performed, with at least two individual spheroids used for each cell line and per condition.

### Colony Formation Assay

A total of 100 μl of culture medium were removed from each well and 100 μl containing 0.6 or 2.8 MBq of ^64^CuCl_2_ were added to 3-day old spheroids. Control spheroids (with 0 MBq) were prepared in a similar way by adding 100 μl of fresh culture medium. After 3 h of incubation at 37°C, at least two spheroids per condition were collected into the same tube, dissociated with Tryple (Gibco) and the number of cells was counted. Dissociated cells were then seeded in T25 flasks: 200 cells in control conditions and 800 for ^64^CuCl_2_-exposed cells. After about 2 weeks of incubation at 37°C, when visible colonies were detected, cells were fixed with a solution of 3:1 methanol:acetic acid at −20°C, washed, and then stained with 4% Giemsa in phosphate buffer at pH 6.8 for 8 min. Colonies with more than 50 cells were counted. Two independent assays were performed. Results are expressed as the % of cellular survival upon treatment when compared with that particular cell line’s untreated control.

### Statistical Analysis

GraphPad Prism 6 software was used to perform all statistical analysis. Data are shown as mean values ± standard error of the mean (S.E.M.) and are the result of at least two independent biological assays with technical replicates as indicated in detail in the above subsections. Statistically significant differences were assessed considering a threshold of *p* = 0.05, using one-way analysis of variance (ANOVA) followed by Dunnett’s test to evaluate the differences between the groups under study.

## Results

### Establishment and Characterization of PCa Spheroids

**FIGURE 1:**
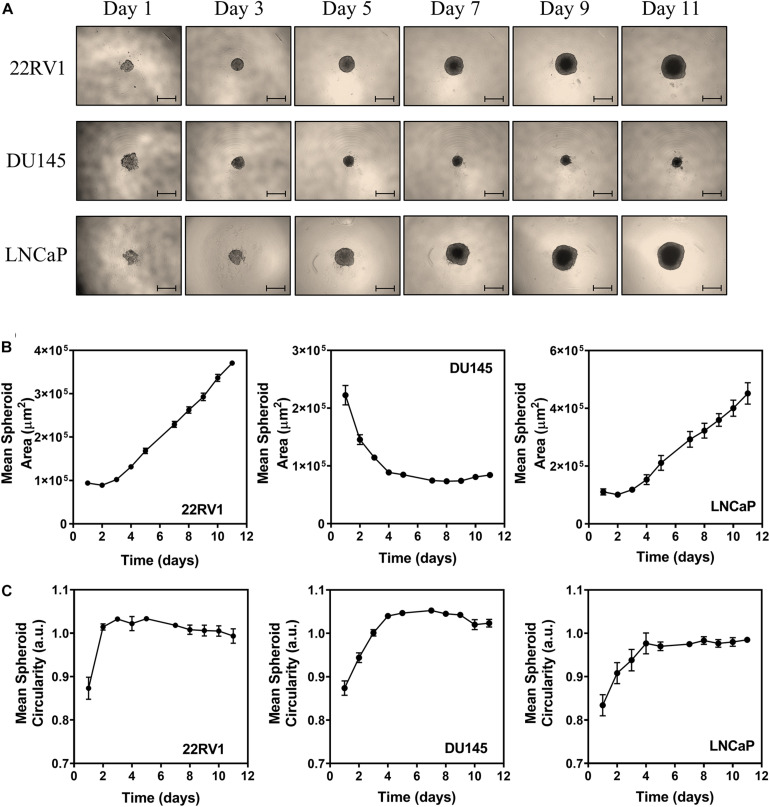
Physical characterization of PCa spheroids. **(A)** Representative microscope images of 22RV1, DU145, and LNCaP spheroids. The images were acquired with a Primovert Inverted Zeiss Microscope (objective 4×). Scale bar, 500 μm. **(B)** Growth curves represented by the mean area (in μm^2^) of 22RV1, DU145 and LNCaP spheroids as a function of the number of days in culture. **(C)** Mean circularity of 22RV1, DU145, and LNCaP spheroids represented as a function of the number of days in culture. Data are presented as the average ± S.E.M. of at least 3 independent assays.

**FIGURE 2:**
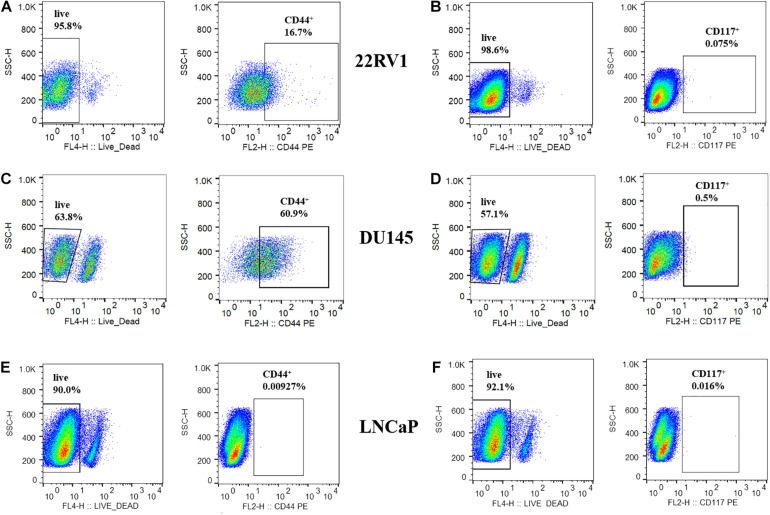
Detection of CD44 and CD117 markers in PCa spheroids by flow cytometry. **(A,C,E)** Population of CD44^+^ cells in 3-day old 22RV1, DU145 and LNCaP spheroids, respectively, gated on live cells. **(B,D,F)** Population of CD117^+^ cells in 3-day old 22RV1, DU145 and LNCaP spheroids, respectively, gated on live cells.

Spheroids from PCa cell lines were prepared using the androgen-independent cell line DU145 and the two androgen-dependent cell lines LNCaP and 22RV1. All cell lines were found to form compact spheroids that were spherical in shape (Figures 1A,C). While 22RV1 and LNCaP spheroids grew in size throughout time, DU145 spheroids exhibited an initial decrease in size that stabilized at around the 5th day of growth (Figure 1B). Since at day three of growth, the spheroids for all cell lines were completely formed with a mean diameter in the range of 350–400 μm, this day was the one considered to start all the studies performed in the following sections. Given that cancer stem cells (CSCs) are known to be involved in treatment failure and cancer relapse (Ishiguro et al., 2017), we also tried to understand if the 3D cellular model used harbored an enriched population of CSCs. For that, we used flow cytometry to evaluate the level of two markers commonly used to characterize CSCs in the PCa spheroids under study (Figure 2): The two markers chosen were CD44 and CD117 since both markers had been described for the identification of CSCs population in PCa (Patrawala et al., 2006; Ma et al., 2012). The percentage of CD44^+^ cells in spheroid-derived LNCaP cells, an androgen-dependent cell line, was irrelevant. On the contrary, DU145, the androgen-independent cell line, expressed the highest percentage of CD44^+^ cells among the PCa cell lines, with the androgen-dependent 22RV1 having a relatively low but clearly noteworthy population of these cells. Regarding CD117, neither 22RV1 nor LNCaP spheroid-derived cells expressed CD117. In contrast, DU145 cells had a very small, but detectable population of CD117^+^ cells. One very surprising result that emerged from this analysis was the observation that the percentage of live cells in spheroid-derived DU145 cells, detected using the LIVE/DEAD assay, was much lower than in cells derived from the spheroids of the two other PCa cell lines.

### ^64^CuCl_2_ Cellular Uptake in PCa Spheroids

**FIGURE 3:**
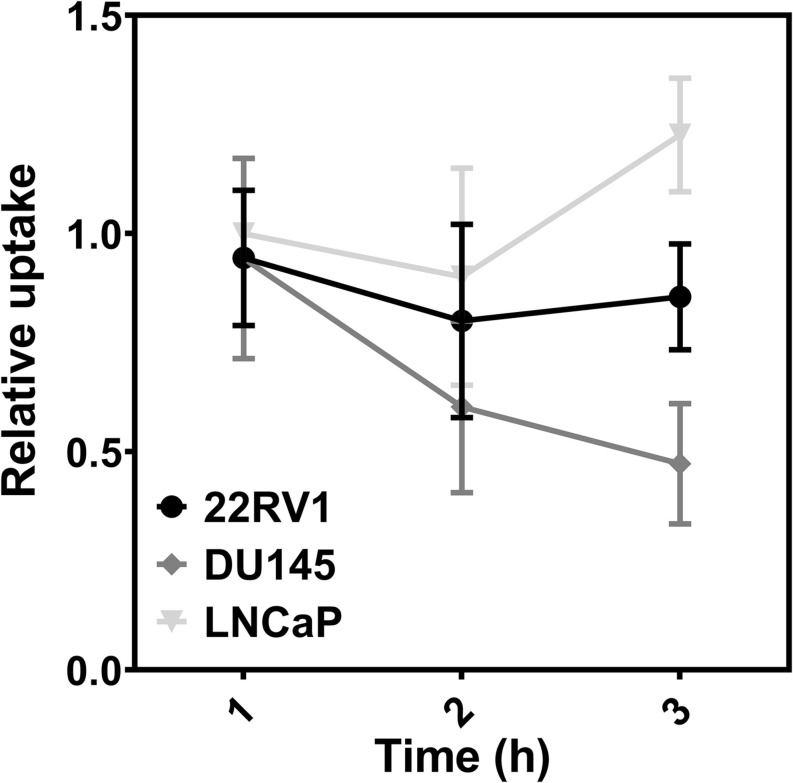
Cellular uptake of ^64^CuCl_2_ in spheroids derived from human PCa cell lines. The cellular uptake was determined at 1, 2, and 3 h after the administration of 0.09–0.19 MBq of ^64^CuCl_2_ and uptake values were normalized to the mean area of the spheroids of each cell line and to the maximum uptake measured at 1 h (in LNCaP spheroids). Data are presented as the average ± S.E.M. of 3 independent assays.

Since 3D culture systems are often more refractory to anti-cancer treatments *in vitro* than 2D culture models due to limited drug penetration (Mittler et al., 2017), we first assessed whether ^64^CuCl_2_ would be taken up by spheroids of three human PCa cell lines: castration-resistant 22RV1 and DU145, and hormone-naïve LNCaP (Figure 3). For that, cellular uptake studies were performed at 1, 2, and 3 h after exposure to ^64^CuCl_2_, in a similar setting to the one used in our previous study done using monolayer-cultured cells (Guerreiro et al., 2018). The results obtained revealed that the spheroids derived from these three cells lines had different behaviors. LNCaP spheroids were the ones that exhibited the highest ^64^Cu uptake, which increased at the last time point analyzed. In contrast, DU145 spheroids, which had the lowest uptake when compared with the other cell lines, exhibited a decrease in uptake over time. 22RV1 spheroids had a stable uptake profile throughout time that was at an intermediate level between the other two cells lines.

### Effects of ^64^CuCl_2_ Exposure on PCa Spheroids’ Growth

**FIGURE 4:**
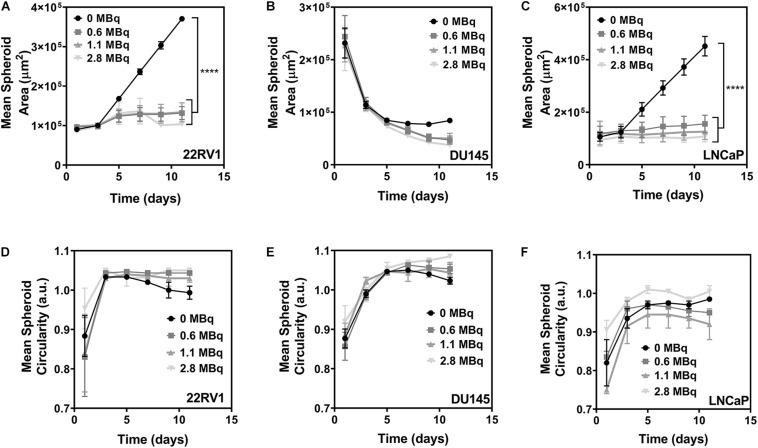
Effects of the exposure to ^64^CuCl_2_ on 3-day old PCa spheroids. **(A–C)** Growth curves represented by the mean area (in μm^2^) of 22RV1, DU145 and LNCaP spheroids, respectively, as a function of the number of days in culture. **(D–F)** Mean circularity of 22RV1, DU145 and LNCaP spheroids, respectively, represented as a function of the number of days in culture. Data are presented as the average ± S.E.M. of 3 independent assays. ****p < 0.0001.

Next, we determined whether the different ^64^Cu uptake profiles exhibited by the PCa spheroids would translate into differences in their growth and shape (Figure 4). An analysis of the evolution of the spheroids′ size over time has revealed that exposure to ^64^CuCl_2_ led to an impairment in the spheroids’ growth for both the 22RV1 and LNCaP cell lines, which exhibited a statistically significant reduction in growth compared to the non-exposed control (Figures 4A,C). This effect was observed shortly after addition of ^64^CuCl_2_ but was seemingly independent from the dose of the radioactive agent used as the magnitude of growth reduction was similar for all the three doses tested. Contrastingly, DU145 spheroids did not exhibit an increase in size even in control conditions as observed for the other two cell lines (Figure 4B). Instead, in control conditions, DU145 spheroids’ size decreased up to the 5th day of culture, after which it stabilized. However, when spheroids were exposed to ^64^CuCl_2_ this decrease in size continued past the 5th day of growth. Even though it is not a statistically significant result, we observed that ^64^Cu-exposed spheroids were clearly smaller than control spheroids at the later time points under study. In addition, some DU145 spheroids showed small cellular deposits around the spheroids, from day 9 onwards, which possibly resulted from partial spheroid disaggregation upon ^64^Cu treatment (Supplementary Figure 1), further indicating that the treatment did have an effect on the spheroids’ integrity.

In terms of the spheroids’ shape, the circularity of 22RV1 and DU145 spheroids (Figures 4D,E) was not severely affected by the treatment, maintaining values close to 1 that indicate that the spheroids were close to being perfectly circular for both cell lines. Differently, LNCaP spheroids presented the circularity values with the highest variation among all the cell lines tested (Figure 4F). This is possibly related with the fact that spheroids from this cell line were by far the most irregular ones in shape even in control condition and, in addition to that, exposure to the radiopharmaceutical also affected the structural integrity of the spheroids. This led to even more irregular shapes and circularity values that were farther from the ones exhibited by non-treated spheroids. This is supported by the observation that, similarly to what was described above for DU145 spheroids, it was possible to observe the formation of a certain precipitate in the wells of LNCaP ^64^Cu-exposed spheroids after the 9th day of growth (Supplementary Figure 1).

### Effects of ^64^CuCl_2_ Exposure on PCa Spheroids’ Viability and Clonogenic Capacity of Spheroid-Derived Cells

**FIGURE 5:**
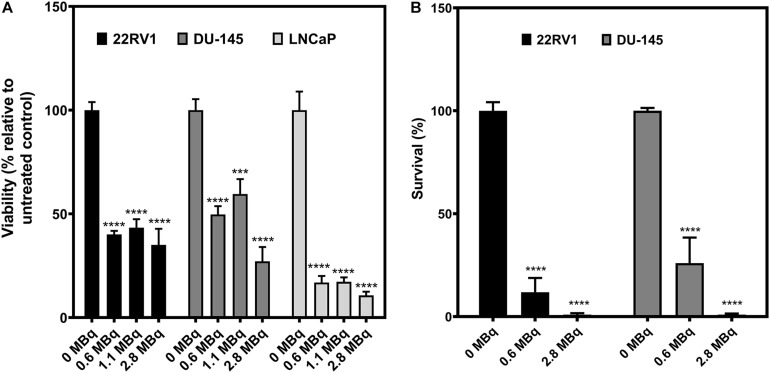
Inhibitory effect of ^64^CuCl_2_ exposure on the viability of PCa spheroids and on the proliferation of spheroid-derived cells. **(A)** Viability of spheroids from human PCa cell lines assessed 8 days after exposure of ^64^CuCl_2_ using the APH assay, and normalized to each cell line’s respective untreated control. **(B)** Survival fractions of PCa spheroids-derived cells after 3 h of exposure to ^64^CuCl_2_, represented as a percentage of the untreated control assessed using the clonogenic assay. Data are presented as the average ± S.E.M. of 3 or 2 independent assays for A and B, respectively. ***p < 0.001, ****p < 0.0001.

After determining that exposure to ^64^Cu had an impact in the spheroids’ growth profile, we went to verify if these changes would be reflected in alterations of the viability and survival of the spheroids (Figure 5). First, the viability of the PCa spheroids was assessed 8 days after exposure to ^64^CuCl_2_ (on the 11th day of growth), using the APH assay (Friedrich et al., 2007; Figure 5A). Without exception, the viability of the ^64^CuCl_2_-exposed spheroids decreased when compared with the non-exposed control spheroids. This was, once more, a dose-independent behavior since all doses used led to a similar reduction in the viability of the spheroids. The only exception to this was found to be the DU145 spheroids that seemed to exhibit lower viability when treated with the highest dose (2.8 MBq of ^64^Cu), compared with the other two doses. Additionally, LNCaP spheroids were the ones presenting the lowest viabilities upon treatment among all the cells lines examined.

To further evaluate the deleterious effects of ^64^CuCl_2_ on PCa spheroids, a clonogenic assay was used to assess the proliferation ability of ^64^Cu-exposed cells in comparison with non-exposed, control cells derived from 22RV1 and DU145 spheroids. Unfortunately, despite our best efforts, it was not possible to perform the assay for LNCaP spheroid-derived cells due to very low platting efficiency (less than 1% in control conditions). The results obtained for 22RV1 and DU145 follow the same trend described above for the viability assay. Both the lowest (0.6 MBq) and the highest (2.8 MBq) dose used led to a very significant decrease in the proliferative ability of spheroid-derived cells, an effect that was common for both cell lines. However, here there was a clear correlation between the dose used and the extent to which the cells’ proliferative ability was affected, with exposure to the highest dose causing a larger reduction in the cells’ clonogenic ability. Also, particularly looking at the results obtained upon exposure to 0.6 MBq of ^64^Cu, the 22RV1 spheroid-derived cells appeared to be the more susceptible to ^64^Cu-induced damage than DU145 spheroid-derived cells.

## Discussion

We have previously demonstrated, using 2D cellular models, that ^64^CuCl_2_ has the potential to have minimal side effects in healthy tissues, while inducing damage in prostate cancer cells (Guerreiro et al., 2018). Importantly, that first proof-of-concept cellular study showed that ^64^CuCl_2_ preferentially entered PCa cells, inducing significant genotoxicity and cytotoxicity in both castration-resistant and hormone-naïve PCa, while sparing non-tumoral cells (Guerreiro et al., 2018). Considering that the findings obtained in the pre-clinical setting are often of limited translational potential due to the use of preclinical models with restricted predictive value (Rodenhizer et al., 2018), in the present work we intended to go beyond the state-of-art by further exploring ^64^CuCl_2_ for PCa theranostic applications using an *in vitro* 3D cellular model that more closely resembled *in vivo* tumors – PCa spheroids. For that, we seeded for each cell line a number of cells that would allow us to obtain 3-day old spheroids with a mean diameter ranging between 350 and 400 μm. This size was chosen taking into account that those spheroids are expected to have a hypoxic core (Ivanov et al., 2014), while still not having a necrotic core (Härmä et al., 2010). This is important because hypoxia is a normal condition in most tumors and contributes to their acquired resistance, while necrosis is undesirable as it might interfere with the cytotoxic measurement of a compound under study (Mittler et al., 2017).

Similarly to what had been seen in monolayer-cultured cells (Guerreiro et al., 2018), spheroid-derived LNCaP cells were the ones found to exhibit the highest ^64^Cu uptake, followed by 22RV1 and DU145 spheroids, the latter being the one that presented the lowest cellular uptake. However, in contrast to what had been observed in 2D cell cultures, spheroids did not show an increase in ^64^Cu uptake throughout time (with the exception of the increase of LNCaP uptake at 3 h), which is coherent with the fact that spheroids have been described as being less permeable to the entry of compounds, due to the establishment of a physical barrier, that is largely created by the existence of tighter interactions among the cells, the presence of ECM proteins, and the interaction between cells and those ECM proteins (Nunes et al., 2019). This physical barrier effect can help to explain the uptake results obtained in our study, since DU145 cells were found to be more compact and much harder to dissociate than the spheroids from the other two cell lines. On the contrary, LNCaP cells formed the less compact spheroids, which might allow for a better penetration of ^64^CuCl_2_ and, consequently, provide the compound with access to a larger number of cells, namely in the innermost layers of the spheroid.

Overall, comparably to what had been observed for monolayer cultures (Guerreiro et al., 2018), exposure to ^64^CuCl_2_ caused a statistically significant decrease in the spheroid growth, viability and clonogenic potential for all cell lines. Notably, spheroids derived from the two androgen-dependent cell lines, LNCaP and 22RV1, were the ones that experienced the highest impairment in growth, while spheroids of the androgen-independent DU145 were the least affected. This trend seemed to persist when considering the results of the viability assay, particularly when looking at the results of the lowest doses of ^64^Cu used (0.6 MBq), with DU145 appearing to be more resistant to treatment with ^64^CuCl_2_, followed by 22RV1 and then LNCaP, which was clearly more susceptible than the other two cells lines. The same profile was found in the clonogenic assay, with DU145 spheroid-derived cells being less affected by the lower dose used when compared with 22RV1 spheroid-derived cells. This resistance profile seems to be in reverse correlation with the uptake profile obtained for the cell lines. It is thus possible that the increased sensitivity demonstrated by LNCaP spheroids was associated with these spheroids taking up more of the radiopharmaceutical. Nevertheless, it is very encouraging to confirm that ^64^CuCl_2_ had a therapeutic effect on PCa spheroids. In addition, this negative effect was largely comparable for the three different doses of ^64^CuCl_2_ used, suggesting that even using a lower dose might be enough to achieve a therapeutic effect, which would lower the burden for patients regarding side-effects.

As mentioned, CSCs have been associated with resistance against anticancer treatments, as well as cancer recurrence (Ishiguro et al., 2017; Nunes et al., 2019). In particular for PCa, CSCs were shown to be androgen-independent, being immune to androgen deprivation therapy (Collins et al., 2005), a characteristic connected with a more advanced stage of the disease that is considered incurable. As such, we tried to understand if the sensitivity demonstrated by the three cell line spheroids could be further explained considering their expression of key markers associated with CSCs. The first marker we selected was the transmembrane glycoprotein involved in cell-to-cell interactions, CD44, since the first PCa CSCs ever isolated were CD44^+^ (Collins et al., 2005), and CD44^+^ PCa cells isolated from tumors were previously reported to be more tumourigenic and metastatic in comparison to CD44^–^ cells, also exhibiting many other stem-like properties (Patrawala et al., 2006). In addition, we also analyzed the levels of the receptor for stem cell factor (SCF) CD117, since the SCF-CD117 signaling pathway has been suggested to contribute to PCa metastization to the bone, as well as to the proliferation and invasion capacities of PCa tumors (Ma et al., 2012). Moreover, PCa cells that overexpress CD117 and ATP-binding cassette super-family G member 2, ABCG2 (CD117^+^/ABCG2^+^), an ubiquitous ATP-binding cassette transmembrane protein that is highly expressed in multi-drug resistant tumor cells, are highly prolific and exhibit multidrug resistance, being able to self-renew and differentiate, as well as to generate tumors *in vivo* similar to the ones from which they were first isolated (Liu et al., 2010). Additionally, PCa tumors that overexpressed CD117 and CD34, a transmembrane glycoprotein ubiquitously used as an hematopoietic cells marker, were also found to be more aggressive and to correspond to a more advanced stage of the disease (Foroozan et al., 2017).

As expected, we observed that spheroids of the cell line that was more affected by exposure to ^64^CuCl_2_, the androgen-dependent LNCaP, were the ones with the lower stemness potential, not expressing neither of the markers tested. In contrast, the androgen-independent DU145 spheroids, which seemed to be the less affected by exposure to ^64^CuCl_2_, were the ones that had a larger population of cells expressing both the markers analyzed. This is in agreement with a previous study that found a large CD44^+^ population in DU145 spheroids, followed by 22RV1 and LNCaP spheroids (Zhang et al., 2012). Contrasting to what was observed with CD44 populations, CD117 was only expressed by DU145 spheroids, unlike what has been reported in the literature for monolayer-cultured cells. Previously, CD117^+^ populations were found to be around 48% in 22RV1 (Liu et al., 2010), 12% in DU145 (Wei et al., 2007) and 20–40% in LNCaP (Liu, 2000) cells. Moreover, another study reported the expression of CD117 in matrigel-embedded spheroids of both 22RV1 and DU145 cell lines, being particularly enriched in DU145-derived spheres (Bahmad et al., 2018). Considering this, it was a bit surprising that we detected such low expression levels of this protein. However, it is known that discrepancies in the expression of markers in certain CSCs, as well as limited reproducibility, can be attributed, for instance, to differences in sample preparation and cellular growth conditions (Nguyen et al., 2012; Moltzahn and Thalmann, 2013). Since the study of CSCs population in spheroid models is not well established and is an evolving field, our results could be expanded by further analyzing a wider panel of markers associated with CSCs in the context of PCa, such as CD133 or $\alpha_{2}\,\beta_{1}$integrin (Collins et al., 2005; Liu et al., 2010). CD133 is a particularly attractive candidate since elevated expression of this cancer stem cell marker and CD44 has been detected in prostate carcinomas, even though the clinical significance of this observation needs to be further elucidated (Kalantari et al., 2017a). Aldehyde dehydrogenase isotype 1A1 (ALDH1A1) is another cancer stem cell marker that has been found to be overexpressed in highly aggressive PCa, suggesting that it might also be of interest to study in this context as a possible target for CuCl_2_-based therapy (Kalantari et al., 2017b).

Strikingly, we found that DU145 spheroid-derived cells had a much lower viability (about ∼30–40% lower) than the cells derived from 22RV1 or LNCaP spheroids. It has been previously described that DU145 spheroids show no growth after formation (Friedrich et al., 2009). Also, when cultured using different matrixes, they present little or no increase in cell growth between the 3rd and the 5th day in culture (Edmondson et al., 2016), which is consistent with what we observed in our growth curves. However, this observation raises the question of whether the decreased uptake of ^64^CuCl_2_ observed for this cell line spheroids could be partly due to a decrease in the number of viable cells. Therefore, the resistance of these spheroids to ^64^CuCl_2_ might be even greater than initially considered because, although having a lower number of viable cells, these were the spheroids showing the most resistant profile. The higher expression of CD44 in DU145 cells might also explain why these cells formed the most compact spheroids, since CD44 is involved in cell-to-cell interactions and cell adhesion (Zhang et al., 2012; Chanmee et al., 2015). Accordingly, LNCaP, forming the least compact spheroids, was negative for this CSCs marker.

While this study has provided important insights into the feasibility of the use of ^64^CuCl_2_ as a theranostic agent for PCa, some questions remain that would be worthy of further investigation. In particular, the ability to analyze the distribution of ^64^Cu in the whole spheroids would be of high interest, since a non-uniform distribution of a radionuclide in a tumor has been shown to have a large impact on its biological effectiveness (Falzone et al., 2018). This is particularly relevant in the case of ^64^Cu, since proximity of the radionuclide to the cellular nucleus is necessary in order for it to be therapeutically effective, due to the short biological range of Auger electrons. In that sense, we are now interested in determining not only the spatial distribution of ^64^Cu, but also to confirm its presence and the induction of DNA damage in the nucleus of spheroid cells, which should allow us to better understand to what extent the compound was able to penetrate the spheroids and target the nucleus. In addition, in the next stage, we are also planning to assess the pharmacokinetics, tumor-targeting ability, and radionuclide therapy potential of ^64^CuCl_2_ in adequate animal models, which will be of high importance to evaluate its real translational potential. Notwithstanding, the results obtained herein put us one step closer towards understanding the mode of action of copper-based radiopharmaceuticals and the development of a potential novel compound for PCa theranostics.

## Data Availability Statement

The original contributions presented in the study are included in the article/Supplementary Material, further inquiries can be directed to the corresponding authors.

## Author Contributions

JG and FM contributed to the conception and design of the study. CP, SB, VA, AF, and JG performed the experiments. AA, CS, and FM contributed to reagents, materials, and analysis tools. CP and JG wrote the first draft of the manuscript. All authors contributed to manuscript revision and have read and approved its submitted version.

## Conflict of Interest

The authors declare that the research was conducted in the absence of any commercial or financial relationships that could be construed as a potential conflict of interest.
