# Supplementary material for: Copper-64 Chloride Exhibits Therapeutic Potential in Three-Dimensional Cellular Models of Prostate Cancer
Source: Front Mol Biosci. 2020 Dec 1;7:609172. doi: 10.3389/fmolb.2020.609172 (PMC7736412; doi:10.3389/fmolb.2020.609172)
Supplement: Supplementary file 1 [file Data_Sheet_1.PDF]

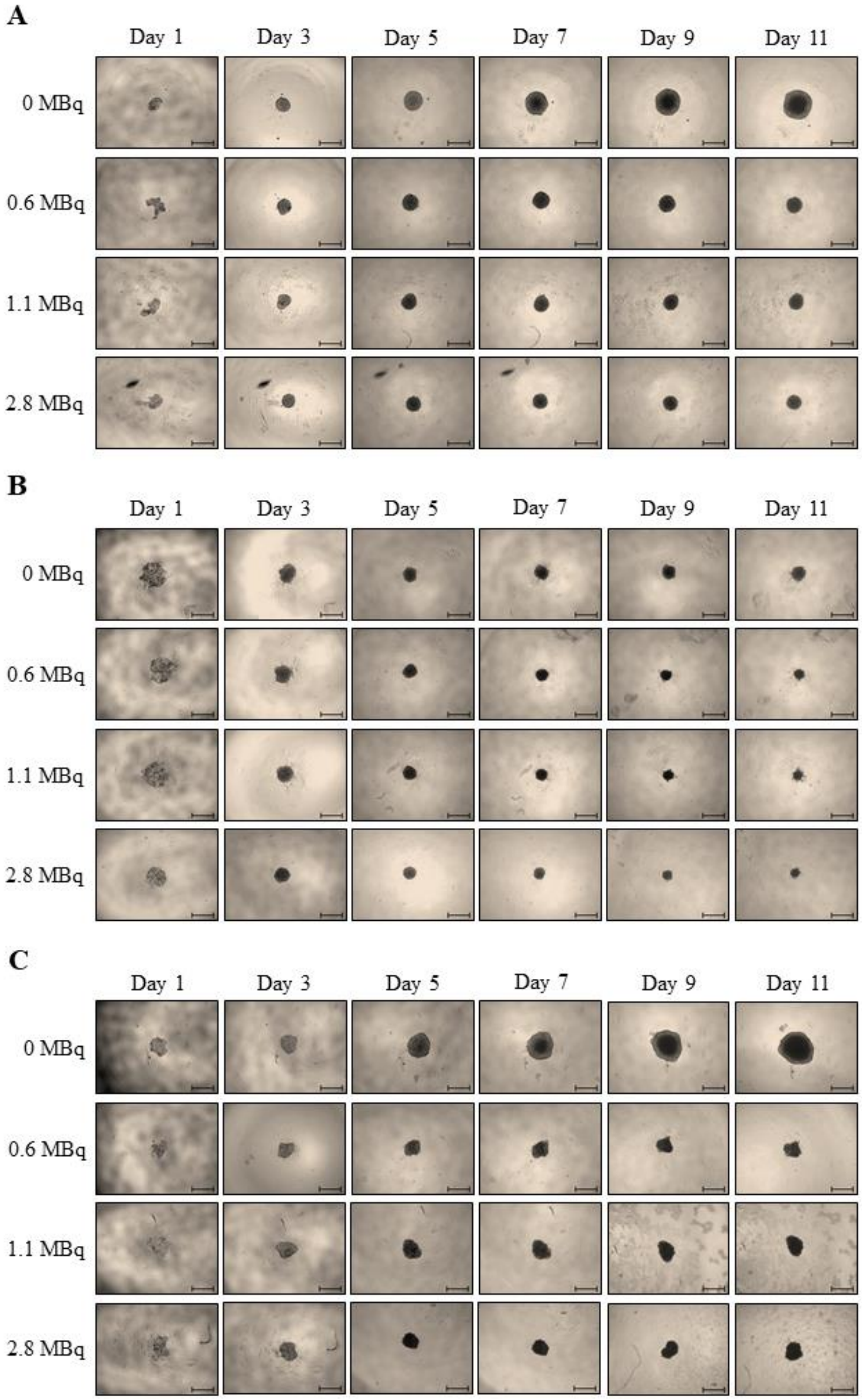

Supplementary Figure 1. Effects of the exposure to $^{64}CuCl_2$ on PCa spheroids. **(A)**, **(B)**, **(C)** Representative microscope images of 22RV1, DU145 and LNCaP spheroids, respectively, after exposure to 0, 0.6, 1.1 and 2.8 MBq of $^{64}CuCl_2$ at day 3 of culture. The images were acquired with a Primovert Inverted Zeiss Microscope (objective 4x). Scale bar, 500 μm.
